# Immune effector cell‐associated haemophagocytic lymphohistiocytosis‐like syndrome

**DOI:** 10.1002/jha2.950

**Published:** 2024-06-16

**Authors:** Enrico Schalk

**Affiliations:** ^1^ Department of Haematology, Oncology and Cell Therapy, Medical Faculty Otto von Guericke University Magdeburg Magdeburg Germany

**Keywords:** cerebrospinal fluid, chimeric antigen receptor T‐cell therapy, cytokine release syndrome, haemophagocytic lymphohistiocytosis, immune effector cell‐associated neurotoxicity syndrome

A 63‐year‐old man with relapsed diffuse large B‐cell lymphoma received autologous anti‐CD19 chimeric antigen receptor (CAR) T‐cell therapy after second‐line treatment and bridging after leukapheresis with rituximab, ifosfamide, carboplatin, and etoposide (R‐ICE). The patient developed cytokine release syndrome (CRS) and further immune effector cell (IEC)‐associated neurotoxicity syndrome (ICANS; immune effector cell‐associated encephalopathy [ICE] score, 9/10), accompanying with peripheral facial paralysis and leg‐pronounced tetraparesis. Treatment with tocilizumab (in total 3004 mg over 4 days) followed by dexamethasone (in total 730 mg over 16 days) was initiated. Magnetic resonance imaging revealed no intracranial pathological findings. On day 11 after CAR T‐cell infusion, cerebrospinal fluid (CSF) analysis showed T lymphocytic pleocytosis (83.6%; almost exclusively CD4^+^); viruses that commonly cause central nervous system infections could not be detected. CD3/CD19 CAR T cells were measured in the CSF with 34.3/μL (compared to 151.7/μL in the peripheral blood). In a cytocentrifuged and May‐Grünwald‐Giemsa‐stained preparation of the CSF many macrophages were seen. Some of them inoculated lymphocytes (Figure 1A; 1000×) or erythrocytes (Figure 1B; 1000×), and are expression of macrophage activation syndrome/haemophagocytic lymphohistiocytosis (HLH). It is most commonly found in the bone marrow but rarely in the CSF. In the context of CAR T‐cell therapy, this toxicity resembling HLH (IEC‐associated HLH‐like syndrome) can manifest as a second inflammatory wave after initial improvement of CRS.

**FIGURE 1:**
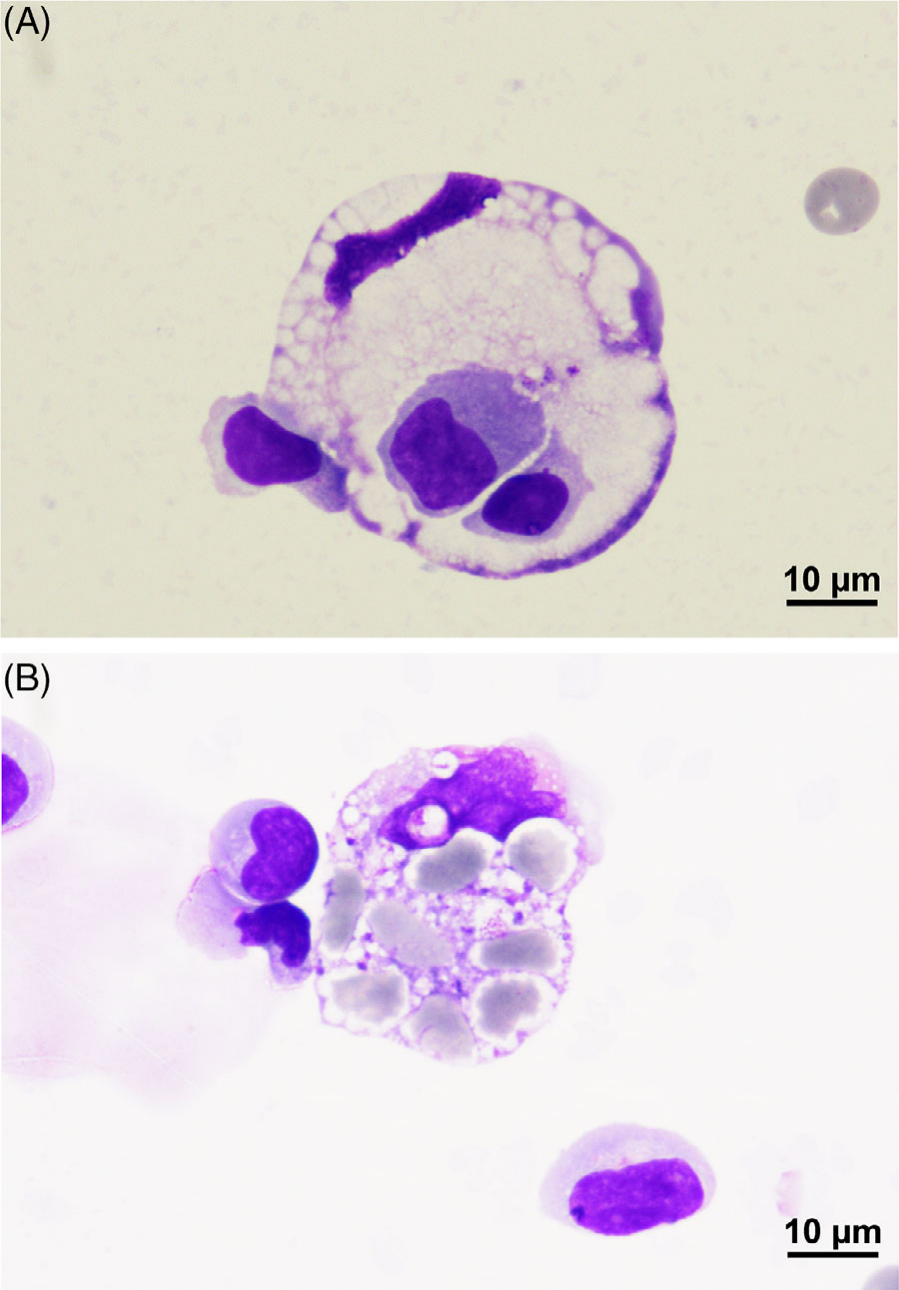
Cerebrospinal fluid. Haemophagocytosis of lymphocytes (A) and erythrocytes (B).

In the further course, the patient developed a second episode of ICANS (ICE score, 9/10) and received methylprednisolone (in total 3000 mg over 3 days), followed by dexamethasone (in total 120 mg over 3 days until discharge). The patient was discharged on day 42 after CAR T‐cell infusion with a tapered dexamethasone scheme. The general condition had improved, but mobility was still limited.

## AUTHOR CONTRIBUTIONS

Enrico Schalk performed the cytological and flow cytometric diagnostics, took the photomicrographs and wrote the manuscript.

## CONFLICT OF INTEREST STATEMENT

The author declares he has no conflicts of interest that are relevant to the content of this article.

## FUNDING INFORMATION

The author received no financial support for the research, authorship, and/or publication of this article.

## ETHICS STATEMENT

The author has confirmed ethical approval statement is not needed for this submission.

## PATIENT CONSENT STATEMENT

The author has confirmed patient consent statement is not needed for this submission.

## PERMISSION TO REPRODUCE MATERIAL FROM OTHER SOURCES

Not applicable.

## CLINICAL TRIAL REGISTRATION

The author has confirmed clinical trial registration is not needed for this submission.

## Data Availability

Not applicable.

